# How does in-store and online shopping experiences influence repurchase intentions in Shandong, China? Roles of perceived value, brand trust, and customer satisfaction

**DOI:** 10.1371/journal.pone.0321485

**Published:** 2025-08-20

**Authors:** Shuang Zhou, Norlaile Salleh Hudin

**Affiliations:** 1 Sultan Idris Education University, Tanjung Malim, Perak, Malaysia; 2 Shandong Liaocheng Zhouxiaobai Biotechnology Co., Ltd., China; Sri Sivasubramaniya Nadar College of Engineering, INDIA

## Abstract

For China’s omni-channel retailers, retaining customers is of utmost importance. This study identifies the key factors influencing customers repurchase intentions in retail stores. Through omni-channel integration, we propose a framework that explains how social media, store atmosphere, merchandise quality, service quality, and price perception impact repurchase intentions through functional, utilitarian, and hedonic value processing systems. The study utilized an online questionnaire targeting 305 respondents, primarily Chinese consumers, considering age, gender, and income levels to enhance methodological transparency. Data validation encompassed factor analysis, confirmatory factor analysis, and structural equation modeling. The results indicate that factors such as social media, store atmosphere, merchandise quality, service quality, and price perception significantly influence perceived value (serving as mediator variables). These perceived values play a crucial role in enhancing customer satisfaction and brand trust. Furthermore, customer satisfaction and brand trust increase repurchase intentions. The findings of this study offer valuable insights for optimizing customer experience in highly competitive markets like China, enhancing loyalty and repurchase intentions, and promoting the success of omni-channel retail strategies.

## 1 Introduction

The global prevalence of new retail represents a significant advancement in democratizing product marketing, surpassing the traditional retail models (e.g., convenience stores and specialty stores) by empowering the public to purchase goods and evaluate content online. New retail involves the innovative transformation of product processes through advanced technologies such as big data and artificial intelligence, integrating online services, offline experiences, and logistics to achieve customer satisfaction. By integrating online and offline resources, enterprises can effectively eliminate barriers between retail channels, breaking down spatial and temporal boundaries for a smoother flow of products, funds, and technology. This integration leads to a positive shopping experience for customers, effectively stimulating their purchase intentions. The superiority of the new retail model lies in its high efficiency and rich experiences, enhancing the customer experience. Consequently, China’s online retail sales reached 15.42 trillion yuan in 2023, marking an 11% increase and solidifying China’s position as the world’s largest online retail market for 11 consecutive years (source: globaltimes). Despite its lucrative nature, the new retail industry remains an underexplored research context, particularly in investigating repurchase intentions [1,2].

The concept of repurchase intention has been studied extensively over the past decades in both academic and practical fields. In physical stores, customer repurchase is the primary goal of retail operators, including many aspects of customer perception of store value, including the image of goods, services, brand, atmosphere, etc. Therefore, the repurchase intention of physical stores is a key research question for retailers. According to data from the Kantar Customer Index, in the first quarter of 2023, the Chinese fast-moving customer goods market resumed growth in urban areas, with a year-on-year increase of 0.3%. Notably, this growth was 4.3 percentage points higher than in the fourth quarter of 2022 (source: kantarworldpanel). In addition, a report jointly released by Bain and Kantar Customer Index indicates that although the value growth rate in the first quarter of 2023 was relatively moderate at 1.9%, it shows that the market is gradually recovering (source: bain).

Customers generally make habitual purchases, shop nearby, or choose the most convenient way for this type of customer goods. Customers do not spend much time thinking or making choices in purchasing decisions. Existing literature extensively studies the concept of repurchase intention in both academic and practical fields, primarily focusing on physical stores where customer repurchase is the primary goal. However, a critical research gap exists in understanding the repurchase intentions within the context of new retail models, especially concerning women’s purchasing behavior for cosmetics. Although prior studies investigate customer purchase intentions through marketing activities such as advertising and salesperson interactions, the real-time interaction between customers and products, particularly in new retail settings, remains overlooked.

With increasingly fierce industry competition and accelerated industry reshuffling, customers’ choices are becoming more rational. Customers are no longer solely concerned about product appearance and cost-effectiveness but also focus more on quality and user experience. From a practical point of view, the insights from this research can help companies analyze the marketing effects of short-term recruitment of new customers, member conversion, and average customer value, as well as long-term repurchase. This enhances their understanding of the impact on retail from a multidimensional and long-term perspective. Thus, investigating women’s willingness to repurchase is crucial in retail, as these insights can enrich supplier knowledge and help determine the attitudes and behaviors of shoppers to improve business operations. From a theoretical perspectives, these findings provide new insights into the role of new retail models and suppliers in promoting repurchase intentions among female customers, which remains an underexplored area in the current literature [3,4].

The objectives of this study are to investigate which tangible and intangible store characteristics impact various aspects of customer behavior within new retail models and to examine the direct impact of these factors on customer behavior and repurchase intentions. Specifically, this research focuses on the shopping experience within retail stores to investigate the future repurchase intentions of retail store customers, with a particular emphasis on women purchasing cosmetics. A three-dimensional research model, including twelve factors, was developed to better decipher the driving factors of customer behavior and repurchase intentions. The study will provide a literature review, summarizing relevant theories and empirical research on customer behavior and repurchase intentions within new retail models in Section 2. Section 3 will describe the research hypothesis used in this study, including the sample selection, data collection, and analysis methods. Section 4 will present the data collection, analysis methods, and findings of the study. Finally, Section 5 and section 6 will conclude the study, summarizing the key findings and implications for both practice and theory.

## 2 Theoretical background

### 2.1 Omni-channel behavior

Omni-channel retailing has garnered significant attention in recent years, particularly as retailers expand their operations across multiple channels [5]. Defined as a marketing strategy where retailers simultaneously employ a portfolio of complementary channels targeting the same customer, omni-channel retailing offers retailers the potential to generate competitive advantages and increase sales revenues by leveraging the strengths and overcoming the limitations of each channel [6,7]. Despite these benefits, the high failure rate of omni-channel operations poses significant challenges. Research has shown that customers who use multiple channels from retailers tend to make more purchases and have higher lifetime value compared to single-channel customers. In response to this trend, an increasing number of online retailers have incorporated mobile storefronts into their channel portfolios. Studies such as Jiang *et al*. [8] have investigated the impact of omni-channel service quality on customer loyalty in both online and mobile retail environments, highlighting the importance of integrating these channels effectively.

Businesses have also leveraged platforms like Ele.me and Meituan Dianping to implement omni-channel marketing models, combining online and offline channels to facilitate customer engagement and increase revenues [9]. The omni-channel model offers customers the convenience of online shopping while allowing them to return to physical stores to check the quality, color, style, and size of goods before purchase [10]. This can lead to cost savings for retailers by reducing last-mile delivery costs and processing returns on-site. However, despite these advancements, the integration between offline and online shopping services for retailers remains relatively weak. Customers have diverse preferences, with some valuing the quality of service provided by merchants and the in-store atmosphere, while others appreciate the convenience of online shopping services [11]. This divide presents a significant challenge for retailers seeking to implement effective omni-channel strategies. To address these issues, this study aims to fill a gap in the existing literature by examining the integration of offline and online shopping services within the context of omni-channel retailing. By identifying and analyzing the specific challenges and opportunities faced by retailers in this area, the study seeks to provide practical insights and guidance for implementing successful omni-channel strategies.

### 2.2 The concept of repurchase intention

Stephani *et al*. [12] defined repurchase intention as the likelihood of customers repeatedly using products or services they had previously purchased. They argued that maintaining customer repurchase intention plays a very important role. Lee [13] described repurchase intention as the likelihood of purchasing individual goods or services again from the same company, mainly based on previous purchasing experience. In cosmetics retail, repurchase intention not only helps businesses save costs but also demonstrates the strength of the brand. Wang *et al*. [14] reported that it is easier for companies to retain existing customers than to acquire new ones. Repurchase intention measures the probability that online customers may purchase from the same online retailer when demand arises. Wijaya *et al*. [15] considered repurchase intention as the likelihood that customers will engage in similar behavior in the future. When online customers are satisfied with an online retailer, they will be loyal and will not consider switching to a different retailer. Yadav *et al*. [16] proposed that customer satisfaction stems from customers’ desires, needs, and expectations that products satisfy, which are indicators of future customer behavior and repurchase intentions. Santoso *et al*. [17] defined repurchase intention as a behavioral factor—namely, a desire and sustained interest in purchasing a product or brand. People are increasingly realizing the need to define repurchase intentions, as they are crucial for maintaining business growth [18]. Furthermore, Luo [19] pointed out that customer behavior occurs when people are stimulated by external factors and then decide to make a purchase. This is based on the customer’s characteristics and decision-making process. Choices regarding brands, products, retailers, quantity, and quality are also factors that influence purchasing decisions. Memon *et al*. [20] concluded that the greater the customer’s familiarity with the product, the higher their confidence and perception of the product, thus increasing their repurchase intention. Wang indicated that brand image is the independent variable, product type is the moderating factor, and purchase intention is the dependent variable. He found that the better the brand image, the higher the customer’s repurchase intention. Zhong [21] found that if a product provides useful features that meet customers’ needs, they will connect with the product and the brand, increasing their purchasing desire.

## 3 Hypotheses development

### 3.1 Conceptual model

**Fig 1:**
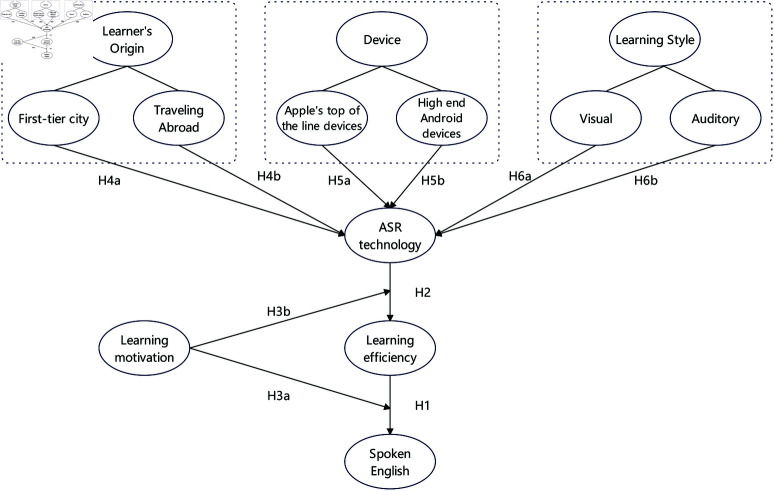
Research model.

This study has carefully designed a research model based on an in-depth analysis of the potential relationships and interactions among various variables. In this model, each arrow is endowed with specific meaning; they are not just graphical connections, but also represent hypotheses and expected relationships in the research. These arrows connect different variables, demonstrating the causal chains and interactions between them, and providing a clear framework and path for subsequent empirical analysis and hypothesis testing. The conceptual model is shown as Fig 1:

### 3.2 Customer satisfaction

Customer satisfaction is the primary goal of any business because it impacts repurchase intention and profit growth [22]. Kumar and Ayodeji [23] emphasized that cumulative customer satisfaction is often a good predictor of customers’ future behavior. Suparna *et al*. [24] observed that emotion-based customer satisfaction (e.g., derived from various experiences) is a more powerful predictor of future behavioral intentions. Similarly, Hartoyo *et al*. [25] argued that the quality of the purchasing experience has a positive effect on customers’ repurchase intentions and that a pleasant experience is related to increased customer support. Chung *et al*. (2019) and Singh *et al*. (2021) suggested that support intention, which includes purchase intention and future preference potential, is the sole indicator that can predict future customer behavior [26]. Similarly, customer repurchase depends on previous positive behaviors and customer satisfaction over time [27]. Rismawati *et al*. [28] concluded that there is a synergistic relationship between customer satisfaction and repurchase intention. Repurchase intention is defined as the customer’s tendency to purchase products from the same manufacturer over an extended period [29]. In addition, understanding the factors that affect repeat purchases provides a sustainable competitive advantage. Hence, the following hypothesis is proposed.

H1: Customer satisfaction positively correlates with repurchase intention.

### 3.3 Brand trust

Brand trust is defined as an emotional investment from customers and a connection between customers and the brand. It is also a powerful influencing factor for various behavioral outcomes. Additionally, brand trust reflects the customer’s dependence on brand performance, which develops after consuming the company’s products. Lee *et al*. (2019) demonstrated that brand trust is an important indicator affecting customer brand commitment, willingness to pay a premium, and customer behavior; that is, brand trust promotes customer purchase intention [30]. Moreover, brand trust can effectively predict customers’ brand purchase intentions, and its predictive validity is greater than that of brand attitude [31]. In the cosmetics industry, female customers are particularly concerned about brand appeal. When customers trust a brand, they are more willing to own its products, leading to repeat purchasing behavior [32]. When customers develop brand trust, they may experience an inner impulse and engage in uncontrolled consumption, known as compulsive buying [33]. Thus, brand trust is considered to have a significant impact on repurchase intention [34]. Hence, the hypothesis is formulated as follows.

H2: Brand trust positively correlates with repurchase intention.

### 3.4 Functional value

Functional value refers to customers’ preferences and evaluations of the functionality, quality, and other aspects of products or services provided by e-commerce websites or retailers. It also reflects the degree to which customer needs and expectations are met. Furthermore, functional value emphasizes the high quality of products or services provided by the website, valuable information content, and high cost-effectiveness. Yan [35], using Huawei smartphones as an example, found that under the mediating effect of customer satisfaction, the relationship between functional value and customer loyalty was significantly positive. The customer’s consumption motivation in functional value lies in the possibility of obtaining corresponding value from consumption behavior and experience. [36]. Functional value includes the search for and updating of knowledge and information obtained by customers [37]. Functional value is driven by customer cognition, reflecting instrumental, functional, and rational aspects [38]. The functional value in this study refers to the perceived experiential value of functionality and practicality that customers gain from using cosmetics, which in turn affects their repurchase intention. Hence, the hypothesis is formulated as follows.

H3: Functional value positively correlates with customer satisfaction.

### 3.5 Utilitarian value

The perceived utilitarian value, perceived functional value, and perceived hedonic value are equally crucial. These attributes form the core reasons for customer choices and continued patronage [39]. Sharma *et al*. [40] stated that perceived utilitarian value reflects the actual benefits that customers derive from a product or service and therefore plays a critical role in customers’ purchasing decisions. According to Grewal *et al*. [41], the customer-brand relationship theory suggests that utilitarian value should enhance brand trust and customer satisfaction. This theory supports the idea that utilitarian value not only fulfills practical needs but also fosters an emotional connection with the brand. As a result, it strengthens brand trust and customer satisfaction. Given the support of these researches, the hypotheses are formulated as follows.

H4: Utilitarian value positively correlates with brand trust.

H5: Utilitarian value positively correlates with customer satisfaction.

### 3.6 Hedonic value

Hedonic value is more subjective than functional value and varies according to individual characteristics [42]. The hedonic value of retail products or services primarily enhances user pleasure and provides spiritual satisfaction. Personalized products may make customers feel better and have a stronger sense of inner satisfaction [43]. Customers derive pleasure from using products or services, enjoy entertainment during the purchasing process, and consider it a pleasant experience [44]. Hedonic consumption is often emotion-driven. Hedonic value is mapped to dimensions based on experience, emotion, and sensory evaluation, expressing experiential emotions related to sensory enjoyment [45]. In addition, hedonic value is a pleasurable experience and sensation directly related to customer emotions and subjective feelings [46]. Hedonic value represents the entertainment benefits perceived by customers, including pleasure and enjoyment [47]. Moreno [48] supported the idea that the purchasing process is a pleasant customer experience. Customers experience entertainment during the purchasing process, which can stimulate customer satisfaction. The hedonic value varies according to customer characteristics and can be mapped to dimensions based on experience, emotion, and sensory evaluation. Customers obtain satisfaction from the use of products or services. Thus, the following hypothesis is constructed.

H6: Hedonic value positively correlates with customer satisfaction.

### 3.7 Social media

Social media is defined as marketing communication through digital applications, platforms, and media, promoting interaction, collaboration, and content sharing among users [49]. Social media has a significant impact on brand reputation [50]. Therefore, organizations and brands should focus on existing SMMA (Social Media Marketing Activities) to attract and attract more customers. In marketing literature, it has been found that the five aspects of SMMA, namely entertainment, interaction, fashion, customization, and WoM (Word of Mouth), significantly affect customer response. Besides, social media interaction involves exchanging information and opinions with other users on social media platforms [51]. The interaction on social media motivates users to communicate with other customers about specific products or brands. Customers often seek various forms of social media to obtain the latest news about specific brands, as it is a reliable source compared to company sponsored marketing communications. Chabuk *et al*. [52] defined fashion as the dissemination of the latest and most trendy information about a specific brand. Social media has a stimulating effect on customer value, such as personal attitudes and subjective norms, or content shared by others on social media. Therefore, when individuals receive and learn the images they see on social media, it will help shape customer attitudes and further influence their perceived value. Thus, the hypotheses formulated as follows.

H7: Social media positively correlates with hedonic value.

H8: Social media positively correlates with utilitarian value.

### 3.8 Store atmosphere

Ali *et al*. [53] define store atmosphere as the long-term summary of customers’ impressions and emotions about store characteristics, as well as other impressions. Store atmosphere includes the “physical environment” and the “in-store environment and layout,” which fall under the scope of environmental psychology theory [54]. This theory posits that the store’s atmosphere significantly affects customers’ emotions and behavioral intentions [55]. A pleasant environment should encourage customers to stay longer in the store, explore the store, and interact with other shoppers or salespeople [56]. Dio *et al*. [57] argue that all physical and tangible cues in a store influence customers’ emotional states and decision-making processes. Delgado *et al*. [58] suggest that store atmosphere is a multidimensional combination of functional and psychological attributes. It represents the overall impression or attitude customers have toward the store. Customers select and evaluate the attributes they consider important from numerous options. Additionally, the store image can change due to factors like time, environment, and purchase experience. The formation of store atmosphere is an interactive process between the customer and the store. The experience and judgment of customers when picking up items in-store will affect their future purchasing behavior. Hence, the following hypotheses are constructed.

H9: Store atmosphere positively correlates with hedonic value.

H10: Store atmosphere positively correlates with utilitarian value.

### 3.9 Price perception

Price perception is defined as the perceived monetary value of a product or service. Price has always been an important driving factor for purchase intention. This is because monetary factors, when purchasing products and services, can affect overall perceived value. Price perception refers to customers’ understanding of the monetary value of products or services. Price has consistently been a significant driver of purchase intention[59]. This is because monetary factors can influence overall perceived value when purchasing products and services. Research shows that price perception not only directly affects customers’ purchase decisions but also indirectly influences their evaluation of product value [60]. Phan Tan and Le [29] found that common promotional strategies, such as bundled promotions, can increase price perception (PPs), thereby stimulating individuals. Aripin *et al*. [61] discovered that common promotional strategies, like bundled promotions, can enhance price perception, which in turn stimulates customers’ desire to purchase. This indicates that when the price is perceived as valuable or attractive, customers are more likely to believe that the product has a higher utilitarian value. Therefore, based on the impact of price perception on purchase decisions and the stimulating effect of promotional strategies on price perception, the following hypothesis is proposed:

H11: Price perception positively correlates with utilitarian value.

### 3.10 Merchandise quality

The concept of merchandise quality is theoretically based on utility theory. According to Cui *et al*. [62], customer behavior depends on the demand for products with desirable attributes. Utility theory posits that customers purchase goods and services to gain certain benefits. When a store offers high-quality products and provides customers with multiple options, it can satisfy their utilitarian motivations [63]. Additionally, research indicates that product quality directly impacts customers’ evaluations of product utility. High-quality goods significantly enhance customers’ perceptions of functional value [64]. According to Zhang *et al*. [65], when a store offers high-quality products and provides diverse choices, it meets customers’ utilitarian motivations. Hence, based on the theoretical and empirical evidence supporting the impact of merchandise quality on customer perception, the following hypotheses are proposed:

H12: Merchandise quality positively correlates with Utilitarian value.

H13: Merchandise quality positively correlates with functional value.

### 3.11 Service quality

The concept of service quality has sparked considerable interest and debate in the research literature [66]. Service quality is usually defined as the degree to which the service meets customer needs or expectations [67]. Therefore, it can be seen as the difference between customer expectations and perceived service. If expectations exceed performance, then perceived quality is unsatisfactory, and customers will be dissatisfied [68]. If merchants are committed to service, are reliable and efficient in communicating with customers, and can solve problems properly, customers will tend to be loyal [69]. Further research indicates that service quality can affect customer loyalty [70]. This finding supports previous research indicating that employee loyalty is significantly correlated with service quality. This, in turn, affects customer satisfaction and loyalty, ultimately leading to improved profitability [71]. Thus, the following hypotheses are formulated:

H14: Service quality positively correlates with utilitarian value.

H15: Service quality positively correlates with functional value.

## 4 Experimental results

### 4.1 Questionnaire development

This study used a quantitative research design to systematically collect data via a questionnaire survey. The questionnaire consists of three parts, designed to explore the impact of retail store shopping experiences on customer behavior. The first part contains items on social media, store atmosphere, price perception, and merchandise quality. These assess the influence of external factors on the customer shopping experience. The second part includes service quality, hedonic value, utilitarian value, and functional value. It aims to evaluate how these service and product characteristics affect customer satisfaction and brand trust. The third part includes customer satisfaction, brand trust, and repurchase intention. It measures customer loyalty and future purchasing intentions. Answering these questions helps identify customers’ feelings and attitudes post-shopping and reveals factors influencing repurchase intention. To ensure validity, the questionnaire items were derived from previous academic research that empirically validated each variable’s scientific and practical relevance. Specific sources are listed in Table 1. The survey was distributed both online and offline, with 400 questionnaires issued to ensure comprehensive data collection. Convenience sampling was employed, targeting retail store customers in Shandong Province. The survey period spanned from January 5, 2024, to May 31, 2024. A total of 305 valid responses were collected. To ensure the representativeness of the sample, the survey targeted consumer groups of varying ages, professions, and monthly cosmetic expenditures. Additionally, questionnaires were distributed at various retail locations and via social media platforms to ensure sample diversity and breadth. Approval from the ethical committee was obtained. All participants provided informed consent before completing the survey. The study strictly adhered to the ethical committee’s guidelines during data collection. All participants signed a consent form prior to completing the questionnaire. The questionnaire was designed anonymously to protect participants’ privacy and data security. The collected data was kept strictly confidential and used solely for academic research, without disclosure of personal information to third parties. The collected data was analyzed using statistical software, including SPSS and AMOS. Descriptive statistics were first applied to summarize the demographic characteristics of the sample. Exploratory factor analysis (EFA) and confirmatory factor analysis (CFA) were performed to examine validity. Structural equation modeling (SEM) was employed to test the hypothesized relationships between the variables.

**Table 1:** Source of questionnaire questions.

Variables	Items	Source
Social Media	4	Pertiwi *et al*. (2020) [31], Connor *et al*. (2020) [51]
Store Atmosphere	7	Marc *et al*. (2019) [55], Caisar *et al*. (2020) [57], Zainuddin *et al*. (2016) [56]
Price Perception	4	Kusumadewi *et al*. (2021) [59], Nurmahdi *et al*. (2023) [85]
Merchandise Quality	5	Bagobiri *et al*. (2022) [68], Choudhary *et al*. (2023) [71]
Service Quality	3	Zhilin *et al*. (2016)[66], Yusriadi, (2023) [4]
Hedonic Value	4	Santini *et al*. (2018) [42], Valentika *et al*. (2021) [47]
Utilitarian Value	3	Zhao *et al*. (2020) [72], Xin, (2023)[21]
Functional Value	4	Haejung *et al*. (2023) [43], Olsen *et al*. (2020) [44]
Customer Satisfaction	3	Chung *et al*. (2019) [22], Balgopal *et al*. (2021) [26]
Brand Trust	3	Zhong, (2023) [21] , Rahmi *et al*. (2021) [32]
Repurchase Intention	3	Wijaya *et al*. (2022)[15] , Yusriadi, (2023) [4]

### 4.2 Basic information distribution

**Fig 2:**
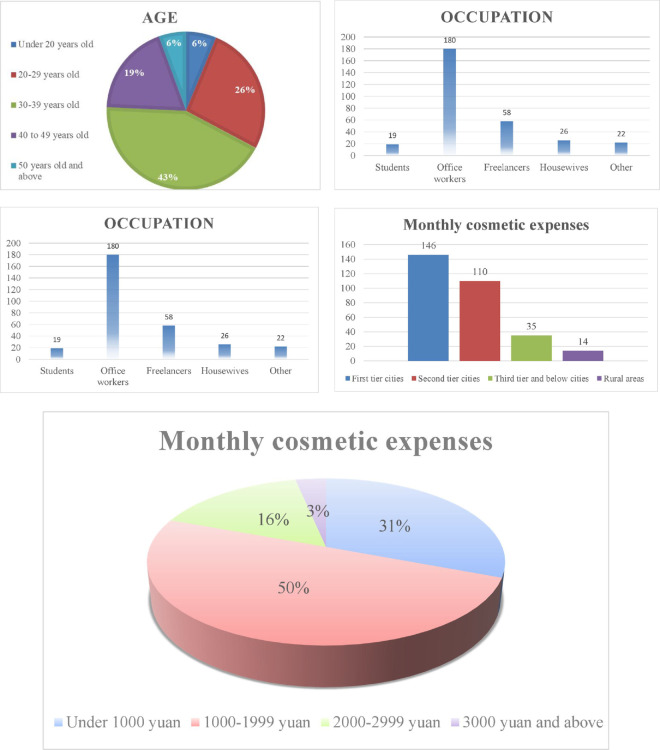
Basic information distribution statistics.

As shown in Fig 2, the largest age group is 30–39 years old, accounting for 43%. The next largest group is 20–29 years old, making up 26%. The 40–49 age group accounts for 19%. Those aged below 20 and those 50 and above are the smallest groups, each at 6%. This indicates that most respondents are between 20 and 39 years old, with these two age ranges combined accounting for 70%. In terms of occupation, office workers dominate the respondents with 180 individuals, far exceeding other occupational groups. Freelancers make up 58 people; housewives, 26; students, 19; and other professions, 22. This suggests that the survey mainly targeted office workers and freelancers. Most respondents have undergraduate or associate degrees. Specifically, there are 144 respondents with a bachelor’s degree, 108 with an associate degree, 34 with high school education or below, 14 with a master’s degree, and only 5 with a doctoral degree. The educational background is primarily concentrated in associate and bachelor’s degrees, indicating a generally high level of education. Most respondents live in first- and second-tier cities. Among them, 146 reside in first-tier cities and 110 in second-tier cities. There are 35 living in third-tier cities or below, and only 14 in rural areas. This shows that the majority of respondents come from urban areas, particularly first- and second-tier cities. Most respondents’ monthly spending on cosmetics is concentrated in the range of 1,000–1,999 RMB, at 50%. The next largest group spends less than 1,000 RMB, at 31%. Those spending 2,000–2,999 RMB account for 16%, and those spending 3,000 RMB or more make up only 3%. This indicates that most respondents have a relatively high spending capacity. In summary, the respondents are primarily well-educated office workers and freelancers living in first- and second-tier cities, aged between 20 and 39, with relatively high monthly spending on cosmetics.

According to Table 2, respondents’ cosmetics consumption is closely linked to factors such as age, occupation, education level, residence, and expenditure. The 30-39 and 20-29 age groups together account for 69% of the respondents. The 30-39 age group, in particular, shows stronger purchasing power and higher demand. Both office workers and freelancers have stable incomes and exhibit higher demand for cosmetics. Office workers tend to have stable expenditures, while freelancers prefer a wider range of brand options. Most respondents are highly educated, prioritize quality and brand, and prefer products with a high cost-performance ratio. Respondents predominantly come from first- and second-tier cities, characterized by strong purchasing power and higher spending. Most respondents spend between 1,000 and 1,999 yuan monthly on cosmetics, indicating both strong purchasing ability and a stable consumption pattern. These factors collectively influence consumers’ purchasing behaviors and decisions.

**Table 2:** Descriptive statistics of basic information.

Category	Item	Frequency	Percentage	Cumulative Percentage
**Age**	Under 20 years old	19	6.2	6.2
	20-29 years old	81	26.6	32.8
	30-39 years old	131	43.0	75.7
	40 to 49 years old	57	18.7	94.4
	50 years old and above	17	5.6	100.0
**Occupation**	Students	19	6.2	6.2
	Office workers	180	59.0	65.2
	Freelancers	58	19.0	84.3
	Housewives	26	8.5	92.8
	Other	22	7.2	100.0
**Education Background**	High school and below	34	11.1	11.1
	Associate degree	108	35.4	46.6
	Undergraduate degree	144	47.2	93.8
	Master’s degree	14	4.6	98.4
	Doctor	5	1.6	100.0
**Residence**	First tier cities	146	47.9	47.9
	Second tier cities	110	36.1	83.9
	Third tier and below cities	35	11.5	95.4
	Rural areas	14	4.6	100.0
**Monthly Cosmetic**	Under 1000 yuan	94	30.8	30.8
	1000-1999 yuan	152	49.8	80.7
**Expenses**	2000-2999 yuan	49	16.1	96.7
	3000 yuan and above	10	3.3	100.0

### 4.3 Factor analysis

#### 4.3.1 Reliability and validity analysis.

In the first part of the factor analysis, we carefully evaluated the reliability and validity of each measurement item in the questionnaire. These evaluations are crucial to ensure that the measurement tools accurately reflect the research concepts. We first focused on the Cronbach’s Alpha coefficient, as shown in Table 3. The overall Cronbach’s Alpha coefficient is 0.932, and each variable’s coefficient is greater than 0.75. This indicates a high degree of internal consistency among the questionnaire items. Generally, a Cronbach’s Alpha coefficient of 0.7 or higher is considered good. Therefore, the high value of 0.932 further confirms the reliability and stability of the measurement tool. In addition, the Kaiser-Meyer-Olkin (KMO) measure is 0.894, indicating the suitability of the sample data for factor analysis. A KMO value of 0.6 or above is generally considered adequate for factor analysis.

**Table 3:** Reliability and validity.

Variables	Items	Alpha	KMO
Social Media	4	0.847	0.816
Store Atmosphere	7	0.922	0.935
Price Perception	4	0.839	0.813
Merchandise Quality	5	0.883	0.871
Service Quality	3	0.801	0.712
Hedonic Value	4	0.855	0.825
Utilitarian Value	3	0.837	0.725
Functional Value	4	0.854	0.815
Customer Satisfaction	3	0.800	0.710
Brand Trust	3	0.832	0.723
Repurchase Intention	3	0.790	0.706
All items	43	0.932	0.894

Therefore, in the reliability and validity analysis, each measurement item exhibits good internal consistency. Additionally, the sample data is suitable to support factor analysis. These results provide a solid foundation for subsequent factor extraction and structural validation, ensuring that the study can effectively explore and explain the relationships among the variables of interest.

#### Factor analysis.

Given the questionnaire’s good internal consistency, this study delved into determining the most suitable number of factors to explain the variable structure. Based on the data, we observed changes in the initial eigenvalues and the sum of squared loadings. The initial eigenvalues indicate the total variance that each factor can explain. The extracted sum of squared loadings shows the percentage of variance and its cumulative percentage for each factor after extraction.

As shown in Table 4, all factors have high initial eigenvalues, explaining a cumulative variance of 71.236%. As the number of factors increases, the eigenvalues of each additional factor decrease, indicating that each factor explains less variance. We also note that the rotated sum of squared loadings is an important measure, especially in interpreting factor structures. By observing the cumulative percentage, we found that all factors cumulatively explained 71.236

**Table 4:** Factor Analysis Results

Item	Initial Eigenvalues			Rotated Loadings Sum of Squares		
	Eigenvalue	Variance (%)	Cumulative (%)	Sum of Squares	Variance (%)	Cumulative (%)
1	11.326	26.339	26.339	4.965	11.547	11.547
2	3.780	8.791	35.130	3.622	8.423	19.970
3	2.440	5.675	40.806	2.864	6.661	26.631
4	2.223	5.169	45.975	2.850	6.628	33.259
5	2.071	4.817	50.792	2.842	6.609	39.868
6	1.877	4.365	55.157	2.744	6.382	46.250
7	1.724	4.010	59.168	2.327	5.412	51.662
8	1.488	3.460	62.627	2.184	5.078	56.740
9	1.316	3.060	65.687	2.160	5.023	61.763
10	1.277	2.969	68.657	2.113	4.914	66.677
11	1.109	2.579	71.236	1.960	4.559	71.236

### Confirmatory analysis

#### Model Indicator Analysis.

Based on the above results, this study delves into various model indicators to evaluate the degree of fit between the model and the data. First, CMIN/DF (chi-square value divided by degrees of freedom) is an important indicator for measuring the goodness of fit between the model and the actual data. As shown in Table 5, in the default model, the CMIN/DF is 1.414. Ideally, this value should be close to 1, indicating that the model may have some shortcomings in interpreting the observed data. Additionally, the NFI, RFI, IFI, TLI, and CFI were all between 0.8 and 0.9, indicating a relatively good model fit. In particular, the IFI, TLI, and CFI are close to 0.9, indicating a high overall model fit. n addition, a GFI above 0.8 indicates that the model fits well. The RMSEA is around 0.03, which is below the threshold of 0.05, indicating a good model fit consistent with the data.

**Table 5:** Model fit of confirmatory factor analysis.

Model Fit	CMIN	DF	CMIN/DF	NFI	RFI	IFI	TLI	CFI	GFI	RMSEA
Fit Results	1180.782	835	1.414	0.839	0.826	0.947	0.942	0.946	0.856	0.037
Judgment Std.			<3	>0.9	>0.9	>0.9	>0.9	>0.9	>0.9	<0.08

In sum, the overall model fit is relatively good based on the evaluation of various indicators from the confirmatory analysis. These results will help further optimize the model’s structure and explanatory power, allowing it to more accurately reflect the complexity of the questionnaire and the factor structures explored in the study.

#### Discriminant Validity and Convergent Validity Analysis.

In confirmatory factor analysis (CFA), discriminant validity is a key indicator for testing whether a construct can be distinguished from other constructs, ensuring that each construct is unique in measurement. To analyze discriminant validity, we compared the square roots of the Average Variance Extracted (AVE) values with the correlations between constructs in the model. To ensure discriminant validity, the square root of each construct’s AVE should be greater than its correlations with other constructs, as shown in Table 6. Convergent validity measures whether the items under the same construct are highly correlated and collectively reflect the construct’s consistency. To evaluate the convergent validity of the questionnaire in this study, we focused on three indicators: standardized factor loadings, CR and AVE. First, by analyzing the standardized factor loadings (as shown in Table 7), we found that all measurement items have factor loadings greater than 0.7. This confirms a strong relationship between the measurement items and their constructs. An AVE greater than 0.50 for all constructs indicates good convergent validity, meaning that more than 50% of the variance is explained by the construct. Most constructs have composite reliability (CR) values above 0.70 and AVE values above 0.50, indicating good reliability and validity. Furthermore, all constructs have AVE values greater than 0.50. This means that constructs explain more than 50% of the variance of their measurement items, indicating good convergent validity. Additionally, the composite reliability values of all constructs exceeded the threshold of 0.70, further verifying the internal consistency of each construct. BBased on these indicators, the measurement model demonstrates good convergent validity, indicating high consistency and correlation among the measurement items under the same construct.

**Table 6:** Discriminant validity.

	SM	SA	PP	MQ	SQ	HV	UV	FV	CS	BT	RI
**Social Media**	0.620										
**Store Atmosphere**	0.380	0.573									
**Price Perception**	0.307	0.175	0.560								
**Merchandise Quality**	0.489	0.241	0.360	0.650							
**Service Quality**	0.344	0.276	0.244	0.301	0.542						
**Hedonic Value**	0.418	0.301	0.398	0.374	0.218	0.559					
**Utiliarian Value**	0.318	0.229	0.190	0.350	0.216	0.270	0.623				
**Functional Value**	0.565	0.369	0.248	0.390	0.304	0.419	0.387	0.700			
**Customer Satisfaction**	0.463	0.303	0.453	0.440	0.321	0.374	0.233	0.462	0.752		
**Brand Trust**	0.544	0.257	0.427	0.451	0.317	0.378	0.232	0.549	0.562	0.775	
**Repurchase Intention**	0.442	0.388	0.337	0.556	0.413	0.303	0.394	0.473	0.462	0.539	0.736

**Table 7:** Convergence validity analysis results.

Construct	Item	Loading Factor	CR	AVE
**Social Media**	SM1	0.730	0.85	0.75
	SM2	0.769		
	SM3	0.739		
	SM4	0.800		
**Store Atmosphere**	SA1	0.731	0.78	0.68
	SA2	0.780		
	SA3	0.799		
	SA4	0.835		
	SA5	0.808		
	SA6	0.787		
	SA7	0.802		
**Price Perception**	PP1	0.781	0.88	0.82
	PP2	0.743		
	PP3	0.765		
	PP4	0.723		
**Merchandise Quality**	MQ1	0.734	0.72	0.61
	MQ2	0.765		
	MQ3	0.768		
	MQ4	0.792		
	MQ5	0.817		
**Service Quality**	SQ1	0.739	0.83	0.73
	SQ2	0.762		
	SQ3	0.762		
**Hedonic Value**	HV1	0.802	0.79	0.69
	HV2	0.786		
	HV3	0.746		
	HV4	0.746		
**Utiliarian Value**	EV1	0.783	0.86	0.77
	EV2	0.815		
	EV3	0.780		
**Functional Value**	FV1	0.735	0.76	0.65
	FV2	0.792		
	FV3	0.756		
	FV4	0.792		
**Customer Satisfaction**	CS1	0.758	0.81	0.71
	CS2	0.723		
	CS3	0.781		
**Brand Trust**	BT1	0.786	0.87	0.79
	BT2	0.804		
	BT3	0.763		
**Repurchase Intention**	BI1	0.787	0.84	0.74
	BI2	0.677		
	BI3	0.725		

#### Structural equation model path analysis.

To gain a deeper understanding of the relationships between latent variables in the research model, we conducted a path analysis using Structural Equation Modeling (SEM). The following are the estimated values, standard errors, critical ratios (C.R. values), p-values, and significance results for each path.

According to the results in Table 8, the path coefficient of social media’s impact on hedonic value is 0.399, which is significant (p < 0.001), indicating that social media significantly enhances customers’ hedonic value. However, the path coefficient of social media’s impact on utilitarian value is 0.137, which is not significant (p = 0.106); therefore, this hypothesis is not supported. The path coefficient of in-store atmosphere’s impact on hedonic value is 0.162, which is significant (p = 0.013), indicating that a good in-store atmosphere can significantly enhance customers’ hedonic experience. However, the path coefficient of in-store atmosphere’s impact on utilitarian value is 0.092, which is not significant (p = 0.175); thus, this hypothesis is not supported. Further analysis shows that the path coefficients of product quality on utilitarian value and functional value are 0.244 (p = 0.002) and 0.372 (p < 0.001), respectively. Both are significant, indicating that product quality has a significant positive impact on both utilitarian and functional values. Similarly, the path coefficient of service quality’s impact on functional value is 0.247 (p < 0.001), which is significant. However, its impact on utilitarian value has a path coefficient of 0.085 (p = 0.250), which is not significant. Additionally, the path coefficients of hedonic value and utilitarian value on customer satisfaction are 0.355 (p < 0.001) and 0.196 (p = 0.004), respectively, both of which are significant. This indicates that both hedonic and utilitarian values have a significant positive impact on customer satisfaction. Finally, the path coefficients of customer satisfaction and brand trust on repurchase intention are 0.272 (p < 0.001) and 0.459 (p < 0.001), respectively, indicating that both significantly enhance customer repurchase intention. Additionally, the path coefficient of functional value on brand trust is 0.555 (p < 0.001), which is also significant. In sum, the path analysis results support most research hypotheses, particularly highlighting the significant impact of social media, in-store atmosphere, product quality, and service quality on customer experience, satisfaction, and repurchase intention. Although utilitarian value did not show significance in all hypothesized paths, its significant impact on customer satisfaction still indicates its important role in customer behavior. These findings further validate the conceptual model proposed in this study, providing valuable insights for retail enterprises to enhance customer experience and increase repurchase intention.

**Table 8:** Structural equation model path coefficient test.

Path	Unstd. Coef.	Std. Error	C.R.	P-Value	Std. Coef.	Conclusion
Hedonic Value ← Social Media	0.395	0.071	5.599	<0.001	0.399	Supported
Utilitarian Value ← Social Media	0.133	0.082	1.616	0.106	0.137	Not Supported
Hedonic Value ← Store Atmosphere	0.159	0.064	2.480	0.013	0.162	Supported
Utilitarian Value ← Store Atmosphere	0.088	0.065	1.357	0.175	0.092	Not Supported
Utilitarian Value ← Price Perception	0.040	0.082	0.493	0.622	0.035	Not Supported
Utilitarian Value ← Merchandise Quality	0.214	0.070	3.063	0.002	0.244	Supported
Functional Value ← Merchandise Quality	0.350	0.063	5.591	<0.001	0.372	Supported
Utilitarian Value ← Service Quality	0.090	0.079	1.151	0.250	0.085	Not Supported
Functional Value ← Service Quality	0.281	0.078	3.614	<0.001	0.247	Supported
Customer Satisfaction ← Hedonic Value	0.339	0.067	5.074	<0.001	0.355	Supported
Customer Satisfaction ← utilitarian Value	0.191	0.066	2.883	0.004	0.196	Supported
Brand Trust ← utilitarian Value	0.099	0.061	1.611	0.107	0.099	Not Supported
Brand Trust ← Functional Value	0.515	0.066	7.813	<0.001	0.555	Supported
Repurchase Intention ← Customer Satisfaction	0.269	0.067	4.004	<0.001	0.272	Supported
Repurchase Intention ← Brand Trust	0.445	0.069	6.461	<0.001	0.459	Supported

### Discussion

**Fig 3:**
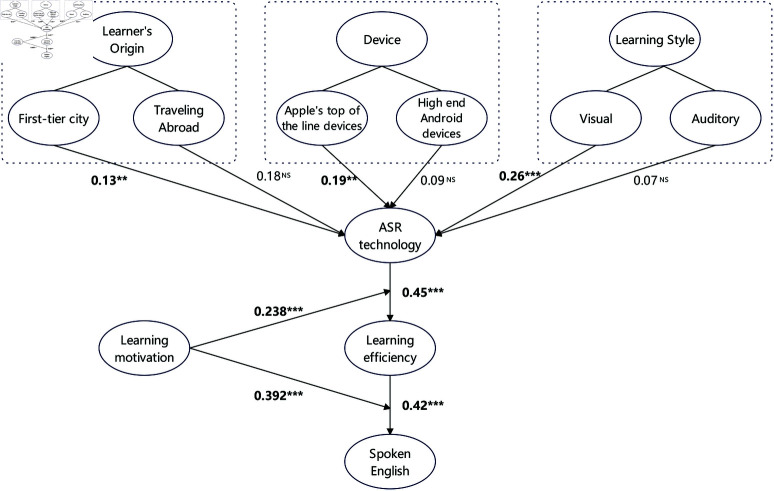
Hypothesis path coefficients.

We conducted an in-depth exploration of the factors affecting the shopping experience in retail stores and validated these factors’ impact on customer behavior through survey data. According to the results in Fig 3 and Table 9, the findings show that customer satisfaction and brand trust have a significant positive impact on repurchase intention, which is highly consistent with the Expectation Confirmation Theory. The Expectation Confirmation Theory states that when customers’ expectations are met or exceeded, their satisfaction and loyalty are significantly enhanced, thus increasing repurchase intention. This validates that customers’ shopping experience and trust in the brand play a core role in driving their future purchase behavior. The theory emphasizes the importance of the practical and economic benefits of products and services on customer satisfaction and trust. This indicates that high-quality internal factors and product characteristics can enhance customer perception of product functionality and utiliarian value, which in turn increases customer satisfaction and brand trust. Additionally, external environmental factors significantly enhance the customer experience of product characteristics, affecting the overall shopping experience and the willingness to repurchase.

**Table 9:** Outcome of the structural model examination.

No.	Hypothesis	Conclusion
H1	Customer satisfaction positively correlates with repurchase intention.	Significant
H2	Brand trust positively correlates with repurchase intention.	Significant
H3	Functional value positively correlates with customer satisfaction.	Significant
H4	Utilitarian value positively correlates with brand trust.	Not Significant
H5	Utilitarian value positively correlates with customer satisfaction.	Significant
H6	Hedonic value positively correlates with customer satisfaction.	Significant
H7	Social media positively correlates with hedonic value.	Significant
H8	Social media positively correlates with utilitarian value.	Not Significant
H9	Store atmosphere positively correlates with hedonic value.	Significant
H10	Store atmosphere positively correlates with utilitarian value.	Not Significant
H11	Price perception positively correlates with utilitarian value.	Not Significant
H12	Merchandise quality positively correlates with utilitarian value.	Significant
H13	Merchandise quality positively correlates with functional value.	Significant
H14	Service quality positively correlates with utilitarian value.	Not Significant
H15	Service quality positively correlates with functional value.	Significant

According to the magnitude of effects, social media has a significant and strong impact on utilitarian value. Social media significantly enhances customers’ hedonic experience, and companies should focus on utilizing social media to boost customer pleasure [73]. In contrast, the impact of social media on hedonic value is limited. Therefore, strategies need to focus on other factors that enhance hedonic value [74]. Furthermore, the store atmosphere has a significant positive impact on customers’ hedonic experience, but the degree of impact is moderate. This shows a progressive relationship compared to the impact of social media. Companies can further enhance customers’ utilitarian value by improving the store environment [75]. On the other hand, the role of store atmosphere in hedonic value is less significant compared to its impact on utilitarian value. Price factors have a weak impact on hedonic value. Companies should consider other more influential factors when adjusting pricing strategies. Merchandise quality plays an important role in enhancing both functional and hedonic value. Companies should prioritize improving product quality to enhance customers’ perception of functional and hedonic value. Service quality plays a significant role in enhancing functional value but has no notable impact on hedonic value. This indicates that improving service quality is especially important for boosting functional value. Customer satisfaction has a strong effect on improving shopping experience and hedonic value. Thus, companies should enhance customer satisfaction to optimize the overall shopping experience. Brand trust has a key role in enhancing functional value but has no significant impact on utiliarian value. This suggests that brand building is crucial for improving functional value. Finally, repurchase intention is significantly influenced by customer satisfaction and brand trust. Enhancing customer satisfaction and brand trust can significantly increase repurchase intention. Both have a notable effect on promoting repurchase intention. Overall, these hypothetical relationships reflect the varying degrees and progressive nature of different factors’ impacts on customer behavior. Companies should consider these factors’ actual impact when developing strategies to effectively enhance customer experience and repurchase intention.

From a broader perspective, customer satisfaction is significantly related to repurchase intention, reflecting the positive impact of customer satisfaction on repurchase intention. When customer satisfaction is high, customer trust in the brand increases. This trust reduces the risk of choice, making them more willing to choose the same brand again and enhancing repurchase intention [76]. Additionally, customer satisfaction reduces search costs. Once customer needs are met, they no longer need to spend time searching for alternatives, leading to direct repurchase and saving decision-making and search time [77]. Highly satisfied customers are more likely to recommend the brand through word of mouth. This word-of-mouth effect not only attracts new customers but also further enhances their own repurchase intention [78]. Customer loyalty increases with satisfaction, and loyalty is an important factor in driving repurchase intention [79]. Satisfied customers are also less sensitive to price changes. Even if the price rises slightly, they are still willing to continue purchasing because they trust the brand to provide high-quality service and resolve issues. For example, Customer A is a 28-year-old advertising professional with high demands for makeup due to a busy work schedule. She often tries new skincare products and follows beauty bloggers, valuing product longevity and ingredients. The salesperson, through professional services, including detailed ingredient explanations and free trials, successfully built her trust in the brand. Customer A’s high satisfaction with the brand makes her more inclined to repurchase in the future and potentially become a loyal customer. Customer B is a young student who mainly focuses on fashion apparel. She shops at a store known for its efficient checkout process and enjoys the quick transactions and personalized service. Customer B’s experience builds on Customer A’s deep experience with the brand, emphasizing shopping efficiency and service quality. Although Customer B’s needs are relatively simple, the efficient service enhanced her loyalty to the brand, making her willing to continue repurchasing. Customer C is a middle-aged professional who values brand reputation and product quality. His lost confidence due to product quality issues but had his trust restored by effective customer service and compensation measures from the brand. Customer C’s experience demonstrates how the brand can further solidify customer loyalty by resolving issues, building on the experiences of Customer A and Customer B. Despite a price increase, Customer C continued to purchase and recommend the brand, highlighting the brand’s crucial role in handling customer issues.

Notably, the impact of price perception on utilitarian value was found to be relatively weak. This non-significant result suggests that while price remains an important consideration for consumers, it may not be the primary driver of perceived utilitarian value in the Shandong market. This could be attributed to various factors such as the economic development of the region, consumer disposable income, and the prevalent pricing strategies employed by retailers. Future research could delve deeper into these potential factors to better understand the nuanced relationship between price perception and utilitarian value. Overall, enhancing customer. satisfaction can significantly boost repurchase intention. This is achieved by increasing brand trust,reducing choice risk, lowering search costs,stimulating word-of-mouth effects, and improving loyalty. Companies should focus on these factors by improving service quality and optimizing the customer experience to enhance repurchase intention and maintain competitive advantage in a highly competitive market. While this study focuses on the Shandong region in China, the findings have broader implications for global retail practice. The integration of online and offline shopping experiences, a trend observed in China, is gradually gaining traction worldwide. Therefore, the insights gained from this study can inform retailers in other countries about the strategic importance of creating seamless, value-laden shopping experiences that resonate with consumers across channels. However, it is crucial to acknowledge potential cultural variations in consumer behavior. Future research should explore how findings from this study might differ in other cultural contexts, considering factors such as individualism versus collectivism, power distance, and uncertainty avoidance. Such cross-cultural comparisons would enrich our understanding of the universality and specificity of shopping experience influences on repurchase intentions.

To further advance our understanding, several specific and actionable research avenues are proposed. Firstly, a deeper exploration of cultural factors could provide insights into the diverse perceptions and valuations of shopping experiences across cultures, potentially involving comparative studies across regions with contrasting cultural orientations to gain a nuanced understanding of cultural moderators. Secondly, longitudinal research is necessary to investigate the dynamic nature of shopping experiences and their long-term impact on repurchase intentions, offering a comprehensive view of evolving consumer preferences and behaviors, thereby enabling retailers to proactively adapt their strategies. Lastly, further investigation is warranted into the role of emerging technologies, such as augmented reality (AR), virtual reality (VR), and artificial intelligence (AI), in shaping shopping experiences, as these technologies rapidly transform consumer interactions with brands and products, potentially influencing perceived value, brand trust, and customer satisfaction in complex ways. Understanding these dynamics is crucial for retailers aiming to remain competitive in the rapidly evolving retail landscape. In conclusion, while this study contributes substantially to our understanding of shopping experience influences on repurchase intentions in Shandong, China, there remains considerable scope for future research to delve deeper into non-significant results, explore global applicability, and propose actionable directions for advancing the field.

## Implications

### Theoretical implications

This study has made significant progress in understanding how customer satisfaction, brand trust, functional value, economic value, and hedonic value shape repurchase intention in retail environments, particularly within the context of new retail models and the cosmetics industry. First, unlike previous research that primarily focused on purchasing behavior or market acceptance, this study delves into the significant impact of customer satisfaction and brand trust on repurchase intention, further validating the importance of customer satisfaction as a crucial predictor of future purchasing behavior, which concurs with the findings of Jiang *et al*. [80]. Second, this study reveals the pivotal role of functional value and practical value in enhancing customer satisfaction and brand trust, echoing the assertions made by Jiandra *et al*. [81] regarding the positive impact of functional value on customer loyalty, emphasizing the indispensable role of product practicality and functionality in building consumer trust and promoting repurchases. Meanwhile, the positive influence of hedonic value on customer satisfaction underscores the importance of emotions and experiences during the shopping process, aligning with the views of Lee *et al*. [82] who emphasized the contribution of a pleasant shopping experience to customer satisfaction and repurchase intention. Furthermore, this study not only deepens the understanding of customer satisfaction and brand trust, especially within the context of new retail models, but also contributes to the knowledge base by demonstrating the positive effects of functional, practical, and hedonic values on customer satisfaction and brand trust. This contribution provides a novel perspective that challenges the limitations of existing literature and underscores the crucial role of optimizing product and service experiences in enhancing consumer satisfaction and loyalty.

### Practical implications

This study provides valuable practical guidance for emerging enterprises facing fierce market competition. Enterprises should prioritize improving customer satisfaction and brand trust, as these two factors have a significant positive impact on repurchase intention [83]. Specifically, enterprises can enhance the functional and utilitarian values perceived by customers by improving product functionality and service quality, such as regularly launching high-quality products, providing personalized services, and conducting customer satisfaction surveys [84]. In addition, research has shown that hedonic value has a significant positive impact on customer satisfaction [85]. Therefore, companies should pay attention to customer emotions and shopping experiences, especially within new retail models. Enterprises can attract and retain customers by creating a favorable shopping environment and providing a pleasant shopping experience. For example, they can create a comfortable atmosphere in physical stores, provide convenient services on online platforms, and use social media to interact with customers by sharing usage experiences and beauty techniques. Through these strategies, enterprises can better meet customer needs, enhance customer satisfaction and brand trust, and thereby occupy a favorable position in the fiercely competitive market, achieving long-term sustainable development. Additionally, businesses should focus on enhancing the functional and hedonic values for customers. For example, by providing high-quality products, offering personalized services, and creating a pleasant shopping environment, businesses can increase customer satisfaction and brand trust. These measures help businesses gain a competitive advantage in a fierce market and achieve long-term sustainable development.

## Limitations and future research

There are some important limitations to this research that deserve attention. First, this study was conducted in a single country, which may limit the generalizability of the findings to other countries due to sociocultural differences [86]. Future research could conduct cross-country comparisons to identify potential differences in the path model for better generalizability. Additionally, efforts should be made to ensure that the sample is representative of the broader population, potentially through stratified sampling or other techniques to address potential biases. Employ random sampling methods to minimize bias and increase the likelihood that the sample accurately reflects the population. Consider using probability sampling techniques, such as simple random sampling, stratified random sampling, or cluster sampling, depending on the study’s requirements. Second, the questionnaire design employed in this research may prevent drawing causal inferences. Future studies should undertake field or laboratory experiments to validate the findings. Third, although this study delineates the relevance of content, hedonic value, utilitarian value, and functional value in stimulating customer internal responses and subsequent customer repurchase intentions, future studies should incorporate other situational factors (e.g., shopping motivation, time pressure, and product class) to further understand customer repurchase intentions. Furthermore, it may be fruitful for future research to examine customer attitudes and behaviors across different livestreaming platforms [87]. Finally, exploring less-charted perspectives (e.g., information exchange and value co- creation) could help understand customer attitudes and behaviors in retail [88]. Lastly, further investigation is needed into how emerging technologies like AR, VR, and AI shape shopping experiences. These technologies are rapidly changing consumer interactions, potentially impacting perceived value, trust, and satisfaction. Understanding these changes is crucial for retailers to stay competitive. These suggestions outline specific directions for future research to address current limitations and expand the knowledge system in this field.

## Conclusion

Given the surging popularity of the Chinese beauty market, brands and retailers must grasp evolving consumer trends to capitalize on this opportunity. This study proposes a comprehensive framework elucidating how customer satisfaction, brand trust, functional value, utilitarian value, and hedonic value influence repurchase intention within the new retail paradigm. Our findings indicate that customer satisfaction and brand trust exert a substantial positive influence on repurchase intention. Furthermore, functional, and utilitarian values are pivotal in fostering customer satisfaction and brand trust. Notably, price perception exhibits a negligible impact on utilitarian value, suggesting that Chinese beauty consumers prioritize factors such as product quality, brand reputation, and personal preference over price. Consequently, brands and retailers should emphasize delivering superior products and services rather than solely focusing on price reductions. Additionally, hedonic value significantly enhances customers’ emotional experiences. These results not only reinforce existing theories, such as Expectation Confirmation Theory and customer value frameworks, but also offer practical insights for domestic cosmetics firms navigating a highly competitive market. By recognizing the significance of functional, utilitarian, and hedonic values in shaping customer satisfaction and brand trust, these companies can refine their marketing strategies to better align with consumer needs. In summary, this study advances our understanding of customer attitudes and behaviors in retail. By improving product quality, optimizing services, fostering a pleasant shopping environment, and leveraging social media, enterprises can bolster customer satisfaction and brand trust, ultimately enhancing repurchase intention and achieving sustainable growth. Moreover, our research underscores the importance of optimizing customer experience and brand management in practice, laying the groundwork for future explorations of customer behavior across diverse cultural and retail contexts, and underscoring the paramount importance of optimizing customer experience and brand management in practical applications, with implications that resonate globally in the dynamic beauty industry. As the retail industry evolves, our findings will contribute to the development of more effective marketing strategies and customer-centric business models.

## Questionnaire questions

**Table:** 

Basic Information	BI1 – Please select your age range:
	BI2 – Please select your gender:
	BI3 – Please choose your profession:
	BI4 – Please select your highest educational level:
	BI5 – Please select your current place of residence:
	BI6 – Please select your average monthly expenditure on cosmetics:
**Social Media**	SM1 – The frequency of brand information updates on social media affects my purchasing decisions for the brand.
	SM2 – I often discuss and share information about cosmetic brands with other users through social media.
	SM3 – I believe that brand activities and interactive content on social media have enhanced my trust in the brand.
	SM4 – I am more inclined to purchase cosmetics brands that have positive reviews and recommendations on social media.
**Store Atmosphere**	SA1 – The interface design and navigation of online stores make me feel happy and convenient.
	SA2 – The product pictures and descriptions in the online store make me more confident in choosing cosmetics.
	SA3 – The virtual makeup test function provided by the online store has improved my shopping experience.
	SA4 – The overall environment and layout of the offline store make me feel comfortable and enjoyable.
	SA5 – The smell and music of offline stores enhance my shopping experience.
	SA6 – The decoration and display of offline stores make me more willing to spend time browsing products.
	SA7 – The cleanliness and tidiness of offline stores have a significant impact on my shopping intention.
**Price Perception**	PP1 – I think the prices of the products in this store are reasonable and worth it.
	PP2 – I think the promotional activities and discounts in this store make the products more attractive.
	PP3 – I usually compare the prices of this store with those of other stores to ensure that I get the best value.
	PP4 – I am willing to pay a higher price for the high-quality products provided by this store.
**Merchandise Quality**	MQ1 – I think the quality of the products in this store is very reliable.
	MQ2 – I am satisfied with the durability of the products in this store.
	MQ3 – I think the products of this store are consistent with their promotional description.
	MQ4 – This store offers customers a variety of choices for its products.
	MQ5 – I think the products in this store are competitive among similar products.
**Service Quality**	SQ1 – I think the service staff at this store are very professional and can effectively solve my problems.
	SQ2 – I am satisfied with the service speed of this store.
	SQ3 – The service staff at this store have a friendly and helpful attitude, which has improved my shopping experience.
**Hedonic Value**	HV1 – I felt very happy and enjoyable trying cosmetics at this store.
	HV2 – The cosmetics display and display in this store made me feel that the shopping experience was very enjoyable.
	HV3 – I like to buy cosmetics at this store because the environment and atmosphere here make me feel relaxed and happy. HV4 – The cosmetics experience activities offered by this store, such as makeup trials and makeup classes, make me feel excited and happy.
**Utiliarian Value**	UV1 – Utiliarian value I think the cosmetics in this store are reasonably priced and have a high-cost performance ratio.
	UV2 – The cosmetics promotion and discount activities at this store make me feel that it’s worth it.
	UV3 – Compared to other brands, I think buying cosmetics in this store is more cost-effective.
**Functional Value**	FV1 – I think the cosmetics in this store have high practicality and functionality, which can meet my needs.
	FV2 – The cosmetics in this store are very effective in use, exceeding my expectations.
	FV3 – The cosmetics ingredients in this store are safe and reliable, which makes me feel at ease when using them.
	FV4 – I think the cosmetics provided by this store can effectively solve my beauty and skincare problems.
**Customer Satisfaction**	CS1 – I am very satisfied with the quality of the cosmetics provided by this store.
	CS2 – I am satisfied with the service attitude and quality of this store.
	CS3 – I am willing to recommend the cosmetics and services of this store to others.
**Brand Trust**	BT1 – I believe that the cosmetics brand in this store can provide stable and high-quality products.
	BT2 – The cosmetics brand of this store makes people feel trustworthy, and I feel confident in its commitment.
	BT3 – I think the cosmetics brand in this store is honest and transparent to customers.
**Repurchase Intention**	BI1 – I plan to continue purchasing cosmetics from this store in the future.
	BI2 – I am willing to recommend cosmetics from this store to my friends.
	BI3 – Even if there are promotional activities from other brands, I will still prioritize the cosmetics in this store.
